# An empirical study on lower limb exoskeleton robots improving athletic performance of disabled athletes through psychological mechanisms

**DOI:** 10.3389/fpsyg.2026.1841408

**Published:** 2026-08-14

**Authors:** Changjian Wang, Yichao Diao

**Affiliations:** 1 Department of Physical Education, Huaibei Institute of Technology, Huaibei, China; 2 Wushu College, Henan University, Kaifeng, China; 3 Graduate School, Kyungil University, Gyeongsan, Republic of Korea

**Keywords:** athletic performance, disabled athlete, lower limb exoskeleton robot, psychological mechanism, self-efficacy

## Abstract

**Objective:**

To explore the improvement effect of lower limb exoskeleton robot intervention on the athletic performance of disabled athletes and the underlying psychological mechanisms, so as to provide empirical evidence and theoretical support for clinical application.

**Methods:**

Thirty disabled athletes with lower limb motor dysfunction were selected for a 12-week randomized controlled trial. Data before and after intervention were collected through athletic performance tests, psychological scale assessments and semi-structured interviews. Statistical analysis was performed using SPSS, and qualitative data were combined to explore the action pathways of psychological mechanisms.

**Results:**

After intervention, the athletic performance indicators of the experimental group were significantly higher than those of the control group; the scores of self-efficacy and intrinsic sports motivation in the experimental group were significantly increased, while the scores of anxiety and depression were significantly decreased, and there was a significant correlation between psychological indicators and athletic performance indicators. Qualitative analysis showed that lower limb exoskeleton robots indirectly promoted athletic performance through four psychological mechanisms: reducing sports frustration, enhancing body control, improving self-identity, and relieving psychological pressure.

**Conclusion:**

Lower limb exoskeleton robots can improve the athletic performance of disabled athletes by improving their psychological state.

## Introduction

With the rapid development of sports for the disabled, improving the athletic performance of disabled athletes has become a research hotspot in sports science and rehabilitation engineering. Lower limb motor dysfunction (such as spinal cord injury, limb deficiency, etc.) is a common functional defect in disabled athletes, which not only limits their sports performance but also easily triggers negative psychological problems such as anxiety, depression and low self-efficacy, forming a vicious circle of “physical disorder—psychological dilemma—decline in athletic performance” (Gao et al., 2025). Existing studies have shown that the psychological state of disabled athletes is closely related to their athletic performance. Psychological factors such as self-efficacy and sports motivation play an important regulatory role in their sports performance, while negative emotions such as anxiety and depression significantly inhibit athletic performance (Xiao, 2013).

As an intelligent rehabilitation device integrating mechanical engineering, electronic technology and biomedicine, lower limb exoskeleton robots can assist lower limb movement through mechanical structures, helping people with lower limb motor dysfunction restore basic motor abilities such as walking and standing, and has achieved remarkable results in rehabilitation medicine (Wang and Zhao, 2024). In recent years, this technology has been gradually extended to the training field of disabled athletes. Existing cases show that disabled athletes wearing exoskeleton robots can complete more complex sports movements and even achieve excellent results in international competitions. For example, Kim Seung-hwan, a Korean disabled athlete, won the championship in the Cybathlon competition wearing the WalkON Suit F1 exoskeleton robot (Zhu et al., 2021); Gianluca Visconti, an Italian disabled athlete, regained confidence in limb movement through exoskeleton experience (Lin et al., 2025). However, current relevant studies mostly focus on the improvement of athletes’ physiological functions (such as muscle activation, gait parameters) by exoskeleton robots, while studies on their indirect improvement of athletic performance through psychological mechanisms are scarce and lack systematic empirical verification (Wang and Zhao, 2021).

Affected by long-term physical disorders, disabled athletes are prone to “learned helplessness,” lack confidence in their sports ability and insufficient sports motivation (Zong et al., 2025). Lower limb exoskeleton robots can help athletes break through physical limitations and complete previously unattainable sports movements by providing mechanical assistance, thereby improving their psychological state. Clarifying the psychological mechanisms through which lower limb exoskeleton robots affect the athletic performance of disabled athletes can provide important references for optimizing training programs for disabled athletes and integrating psychological and technical interventions. Meanwhile, it promotes the standardized application of lower limb exoskeleton robots in sports for the disabled and helps disabled athletes realize their sports and self-values.

At present, academic research on the application of lower limb exoskeleton robots in disabled athletes mainly focuses on three aspects: first, the physiological level, exploring the improvement effect of exoskeleton robots on lower limb muscle strength, gait parameters and muscle activation patterns of athletes. For example, Zhu et al. (2021) found that wearing exoskeleton robots can significantly affect muscle activation and gait parameters and reduce muscle fatigue (Gu et al., 2025). Second, the psychological level, some studies found that exoskeleton robots can improve self-identity and quality of life of disabled athletes and alleviate negative emotions (Liu et al., 2024). Third, the design and simulation of lower limb exoskeleton rehabilitation robots (Zhang and Ning, 2025). On the whole, current research is in-depth and extensive, but there are also deficiencies: first, most studies focus on physiological improvement and pay less attention to psychological impact; second, lack of special empirical research targeting disabled athletes, mostly extended from research on rehabilitation patients, which cannot fully reflect the sports needs and psychological characteristics of disabled athletes; third, existing studies have not clarified the psychological mechanisms of exoskeleton robots affecting athletic performance, and research on the psychological characteristics of disabled athletes is relatively lagging behind; fourth, existing studies are mostly qualitative or small-sample exploratory studies, lacking large-sample and long-term intervention empirical research, and studies combining exoskeleton robots, psychological mechanisms and athletic performance to systematically explore their internal correlation are also scarce.

There are four major research gaps in the current system: First, the research subjects are mismatched. Most studies on the psychological effects of exoskeletons focus on general rehabilitation populations like stroke or spinal cord injury patients, ignoring the unique psychological traits of professional disabled athletes, such as long-term competitive training, competition pressure, and specific sports needs. Second, the variables are disconnected. A coherent conceptual framework has not been established, and the chain of relationships between exoskeleton use, technology acceptance, self-efficacy, intrinsic sports motivation, anxiety and depression, and sports performance has not been systematically clarified. Third, there are methodological flaws. Existing studies mostly involve short-term observations or small-sample qualitative interviews, lacking mixed-method empirical research such as 12-week standardized randomized controlled trials or mediation effect quantification. Fourth, there is an application gap. The specific subjective experience dimensions through which exoskeletons provide psychological benefits have not been clearly defined, making it impossible to offer a replicable practical basis for an integrated ‘equipment training + psychological guidance’ training program.

Based on the research gaps mentioned above, this study conducted a 12-week randomized controlled mixed-methods trial. The main motivations are: (1) to quantitatively verify the overall improvement effects of lower-limb exoskeletons on the athletic performance and multidimensional psychological indicators of professional athletes with disabilities; (2) to build a coherent mediation concept model to clarify through which psychological pathways the exoskeleton indirectly enhances athletic performance; (3) to refine athletes’ subjective psychological experiences and improve theories of sports psychology for people with disabilities in human-machine interaction; (4) to develop a repeatable, standardized exoskeleton training intervention process, providing empirical guidance for Paralympic training and adaptive sports promotion. This study uses quantitative statistics and qualitative interviews in a triangulated approach to make up for the limitations of current single-perspective research, filling an empirical gap at the intersection of smart assistive devices, sports psychology, and athletic performance.

Therefore, this study adopted a randomized controlled trial design, selecting disabled athletes with lower limb motor dysfunction as research subjects, divided into experimental group and control group, receiving lower limb exoskeleton robot training and conventional rehabilitation training respectively, with an intervention period of 12 weeks. Through athletic performance tests and psychological scale assessments before and after intervention, relevant data were collected. Statistical analysis was used to compare the differences in athletic performance and psychological state between the two groups, and to explore the mediating role of psychological mechanisms between lower limb exoskeleton robots and athletic performance. Meanwhile, combined with semi-structured interviews, athletes’ subjective experiences were explored to further verify the action pathways of psychological mechanisms. Theoretically, this study aims to enrich the theoretical system for improving athletic performance of disabled athletes, reveal the internal correlation between lower limb exoskeleton robots and psychological state and athletic performance of disabled athletes, improve the theoretical basis for the application of exoskeleton robots in sports, and enrich the interdisciplinary theory of sports psychology and rehabilitation engineering. Practically, it provides new ideas and methods for the training of disabled athletes, empirical support for the popularization and application of lower limb exoskeleton robots in sports for the disabled, and targeted strategies for psychological intervention of disabled athletes, helping them perform their best in sports competitions and achieve self-breakthrough.

### Theoretical analysis and research hypotheses

The theoretical basis of this study mainly relies on self-efficacy theory, self-determination theory and stress coping theory, clarifying the internal correlation among lower limb exoskeleton robots, psychological mechanisms and athletic performance.

Self-efficacy theory was proposed by Bandura. This theory holds that self-efficacy is an individual’s judgment and confidence in his ability to complete a certain behavior, and its level directly affects the individual’s behavior choice, effort level and persistence (Han et al., 2025). For disabled athletes with lower limb motor dysfunction, long-term physical limitations tend to lead to low self-efficacy, thus daring not to challenge sports goals, lacking training persistence, and ultimately affecting athletic performance. Lower limb exoskeleton robots compensate for physical defects through mechanical assistance, helping athletes complete previously unattainable sports movements and gradually accumulate successful experiences, thereby improving self-efficacy and laying a psychological foundation for the improvement of athletic performance. This is highly consistent with the core view of self-efficacy theory that “successful experience is the core way to improve self-efficacy.”

Self-determination theory points out that an individual’s intrinsic motivation is the core driving force for his behavior. When an individual’s needs for autonomy, competence and belonging are met, intrinsic motivation will be significantly enhanced (Yue et al., 2021). Due to physical disorders, disabled athletes are difficult to gain a sense of competence in conventional training, prone to passive training and insufficient intrinsic sports motivation. The assistance of lower limb exoskeleton robots can help athletes break through physical limitations, feel the improvement of their sports ability and meet the need for competence; meanwhile, the personalized adaptation mode of robots respects athletes’ training wishes and meets the need for autonomy, thereby stimulating intrinsic sports motivation, promoting their active participation in training and improving athletic performance.

Stress coping theory believes that when individuals face stressors, they will form corresponding coping styles through self-regulation, and negative emotions (such as anxiety, depression) will reduce individuals’ coping ability and thus affect behavioral performance (Mu, 2022). Physical disorders of disabled athletes are long-term stressors, which easily trigger negative emotions such as anxiety and depression and inhibit the performance of sports ability. As an effective stress relief carrier, lower limb exoskeleton robots help athletes relieve psychological pressure caused by physical disorders and reduce negative emotions by improving physiological functions and athletic performance, thereby improving athletic performance. This is consistent with the view of stress coping theory that “positive coping styles can relieve stress and improve behavioral performance.”

In addition, combined with the Technology Acceptance Model (Hu et al., 2022), as an advanced rehabilitation training technology, the usability and effectiveness of lower limb exoskeleton robots affect athletes’ acceptance. The higher the acceptance, the higher the athletes’ training participation, thereby indirectly affecting athletic performance through the improvement of psychological state, which conforms to the theoretical logic of “technical intervention—psychological regulation—athletic performance.”

This study establishes a chain mediation framework: lower-limb exoskeleton-assisted training → increased acceptance of the technology → improvement in multi-level psychological variables → enhanced athletic performance. The expected outcomes are as follows:

1. Exoskeleton intervention → technology acceptance: The anthropomorphic gait, adaptive wear, and real-time motion feedback of the lower-limb exoskeleton enhance perceived usefulness (PU) and perceived ease of use (PEOU). According to the Technology Acceptance Model, higher device acceptance boosts athletes’ willingness to continue training and their engagement in human-machine collaboration. Lorenzutti et al. (2025) confirmed that exoskeletons can reduce cognitive load during exercise and ease anxiety about device operation, directly increasing long-term usage intention, laying the foundation for subsequent psychological benefits.

2. Technology acceptance → positive psychological capital (self-efficacy, intrinsic exercise motivation):

(1) Self-efficacy: Continuous use of the exoskeleton enables completion of walking and lower-limb control movements previously impossible, accumulating successful experiences, which is a core approach to boosting exercise self-efficacy. Pinto-Fernandez et al. (2020) found that lower-limb exoskeleton training can reshape the body schema perception of spinal cord injury patients, reduce discomfort, and significantly enhance confidence in physical abilities, matching the expected path of this study.

(2) Intrinsic exercise motivation: Personalized power adaptation of the exoskeleton satisfies athletes’ autonomy needs; mechanical assistance overcomes physiological limits and allows high-difficulty movements to meet competence needs; stable long-term training helps form a sense of group belonging, meeting attachment needs. Once these three basic psychological needs are fulfilled, externally imposed training transforms into intrinsic exercise motivation.

3. Technology acceptance → alleviation of negative emotions (anxiety, depression): According to stress coping theory, lower-limb dysfunction is a persistent stressor for disabled athletes, with long-term stress inducing somatic anxiety and exercise-induced depression. Exoskeletons reduce the likelihood of training failures and enhance movement stability, providing effective active stress-coping resources. Babič et al. (2021) noted that upright walking and independent movement with an exoskeleton improve users’ social self-esteem, reduce psychological trauma and low mood due to disability, significantly lowering SAS and SDS scores.

4. Increased positive psychology and decreased negative emotions → improved athletic performance: Anderson et al. (2025) showed that high self-efficacy and intrinsic motivation enhance training persistence and willingness to attempt difficult movements, optimizing attention allocation. Anxiety and depression consume cognitive resources, disrupt gait control, and reduce endurance, creating a bidirectional psychological impact on athletic performance.

5. Expected Chain Mediation Paths: Lower-limb exoskeleton-assisted training → higher device acceptance (perceived usefulness / ease of use) → Path 1: boost exercise self-efficacy → improve walking speed, stride length, endurance, and movement accuracy → Path 2: trigger intrinsic exercise motivation → improve walking speed, stride length, endurance, and movement accuracy → Path 3: reduce exercise anxiety → improve walking speed, stride length, endurance, and movement accuracy → Path 4: ease depressive feelings → improve walking speed, stride length, endurance, and movement accuracy.

Based on the above theoretical analysis, the following research hypotheses are proposed:

*Hypothesis H1*: Compared with conventional rehabilitation training, lower limb exoskeleton robot training can significantly improve the athletic performance (walking speed, step length, sports endurance, sports accuracy) of disabled athletes;

*Hypothesis H2*: Compared with conventional rehabilitation training, lower limb exoskeleton robot training can significantly improve the psychological state of disabled athletes (improve self-efficacy, enhance sports motivation, relieve anxiety and depression);

*Hypothesis H3*: Lower limb exoskeleton robots indirectly improve the athletic performance of disabled athletes by improving their psychological state (self-efficacy, sports motivation, emotional state), and psychological mechanisms play a mediating role.

## Research subjects and methods

### Research subjects

#### Screening of research subjects

Participant Recruitment: This study uses multi-channel purposive sampling. Recruitment announcements were posted uniformly through the city-level Disabled Sports Association and the sports rehabilitation departments of rehab hospitals, clearly stating the study period, training content, equipment type, testing procedures, and potential benefits along with minor risks like muscle soreness and discomfort from wearing the equipment.

Thirty disabled athletes with lower limb motor dysfunction were selected as research subjects. The inclusion criteria were: aged 18–45 years, regardless of gender; lower limb motor dysfunction (spinal cord injury, limb deficiency, etc.), medically diagnosed as moderate dysfunction, able to sit independently, with basic cognitive and communication abilities; more than 1 year of sports training experience, participated in municipal and above disabled sports competitions; no serious heart, liver, kidney and other organ diseases, no mental illness history, no contraindications to wearing exoskeleton robots; voluntarily participated in this study, able to cooperate with 12 weeks of intervention training and various tests, and signed informed consent.

Exclusion criteria: serious injuries, equipment discomfort and other conditions during intervention, unable to continue training; failed to complete training and tests as required, with serious data loss; received other rehabilitation treatment or psychological intervention during intervention, which may affect the research results.

#### Basic information of research subjects

Thirty research subjects were randomly divided into experimental group and control group by random number table method, with 15 subjects in each group. In the experimental group, there were 9 males and 6 females; aged 22–43 years, with an average age of (32.5 ± 5.8) years; 8 cases of spinal cord injury and 7 cases of limb deficiency; sports included track and field (6 cases), swimming (4 cases), table tennis (3 cases) and others (2 cases). In the control group, there were 8 males and 7 females; aged 21–44 years, with an average age of (33.2 ± 6.1) years; 7 cases of spinal cord injury and 8 cases of limb deficiency; sports included track and field (5 cases), swimming (5 cases), table tennis (3 cases) and others (2 cases). There were no statistically significant differences in age, gender, disability type, sports and other basic data between the two groups (*p* > 0.05), which were comparable.

#### Ethical approval and participant protection

This study protocol was approved by the Ethics Review Committee of Huaibei Institute of Technology (No. 2025LL017) and strictly follows the Declaration of Helsinki. All participants signed a paper informed consent form, which included: the purpose of the study, a 12-week training schedule, test items, data confidentiality rules, the right to withdraw at any time without conditions, emergency plans for adverse events, no additional financial compensation, and the option to use the exoskeleton for free for 4 weeks after training as an incentive. All collected data were anonymized, with codes replacing names; motion capture videos and interview recordings were only used for this study and will be destroyed 12 months after the study ends. The training was fully supervised by rehabilitation physicians and sports protection specialists, and training was stopped immediately if pain or dizziness occurred.

### Research methods

#### Experimental design

A randomized controlled trial design was adopted, with an intervention period of 12 weeks, 5 training sessions per week, 60 min per session. The training intensity and content (except intervention methods) of the two groups were consistent. The experimental group received lower limb exoskeleton robot-assisted training, and the control group received conventional rehabilitation training (such as elastic bands, balance boards, rehabilitation balls, etc.) for lower limb muscle strength training, balance training, joint mobility training, passive and active lower limb exercise and other conventional rehabilitation programs. The training program was formulated by professional rehabilitation therapists according to the specific conditions of athletes. One week before intervention, baseline tests (athletic performance, psychological state) were conducted on both groups; mid-term tests were conducted at the end of the 6th week of intervention; final tests were conducted at the end of the 12th week of intervention, and relevant data were collected for analysis. Meanwhile, after the intervention, semi-structured interviews were conducted with athletes in the experimental group to explore their psychological experience and subjective feelings.

#### Randomization and blinding plan

Sequence Generation: An independent statistician used SPSS 26.0 to generate 30 random numbers. Numbers 1–15 were assigned to the experimental group and 16–30 to the control group, using fixed block randomization (block size 6) to ensure the two groups were balanced 1:1.

Allocation Concealment: The random sequence was sealed in opaque envelopes. After all baseline tests were completed, a third-party staff member, who did not participate in recruitment or testing, opened the envelopes to assign groups. Researchers could not predict group assignments in advance.

Blinding: Due to differences in intervention devices, participants and rehabilitation trainers could not be blinded. Outcome assessors and data analysts were blinded throughout the study. Assessors did not interact with the training process and performed gait and scale assessments based only on participant numbers. Group labels were hidden during data analysis.

#### Intervention plan, standardized training progress, and compliance management

Intervention Duration and Training Arrangement: The total intervention period is 12 weeks, divided into an adaptation phase (weeks 1–4), an enhancement phase (weeks 5–8), and a consolidation phase (weeks 9–12). Training is scheduled 5 times per week, each session lasting 60 min, held uniformly from 14:00 to 15:00. Both groups have identical training loads and warm-up/cool-down routines, with only the core intervention tools differing.

Warm-up 10 min: dynamic stretching, joint mobility;

Core Training 40 min (Experimental group: Aider3S exoskeleton-assisted gait, balance, and lower limb strength training; Control group: conventional lower limb rehabilitation with resistance bands, balance board, and rehab ball);

Cool-down 10 min: static stretching, foam rolling.

#### Progressive training schedule

Weeks 1–4 (Adaptation): Low-intensity gait practice, walking distance ≤200 m per session, focusing on getting familiar with equipment and human-machine coordination rhythm;

Weeks 5–8 (Enhancement): Add variable speed walking, turning, and step simulations, walking distance 300–500 m per session, include precise lower limb positioning exercises;

Weeks 9–12 (Consolidation): Combine multiple movement tasks, continuous 6-min walking, complex directional changes, simulate on-court movement scenarios.

#### Compliance management and dropout handling

Attendance system: Sign in for each session, therapists record attendance and completed training volume; establish a WeChat check-in group with daily training reminders.

Dropout criteria: Missing ≥2 sessions in a week or ≥8 sessions overall counts as dropout, and data is excluded.

Compliance statistics: All 30 participants completed the study with no dropouts, overall compliance 100%; completed all three tests at baseline, 6-week midpoint, and 12-week endpoint; all interviews completed with no missing recordings.

#### Intervention tools

Lower limb exoskeleton robot: A lower limb walking exoskeleton robot (Chengdu Buffalo Robot Technology Co., Ltd., Model Aider3S) was selected. This equipment has been iterated for many generations, can be worn in 60 s, gait parameters can be automatically adjusted according to user habits, and has multi-mode training functions such as active, passive and isokinetic, which can simulate anthropomorphic gait, help users correct stepping posture and rebuild walking memory of nerves and muscles (Huo et al., 2023). The equipment is equipped with sensors to monitor motion data in real time to ensure the safety and adaptability of training. Its core technology conforms to ergonomic design, which can minimize wearing discomfort and is suitable for long-term training of disabled athletes.

#### Test indicators and tools

(1) Referring to existing studies (Wei et al., 2008; Pelletier et al., 1995; Zung, 1971; Zung, 1965), the athletic performance test indicators shown in Table 1 were selected:

**Table 1:** Test indicators.

Test metrics	Test method	Reasons for choosing	Source
Walking speed	Test the athlete’s average speed walking 10 meters in a straight line, in units of m/s, test three times, and take the average value.	The core movement limitations of athletes with lower limb disabilities are concentrated on walking ability, gait stability, and endurance levels. Step speed and stride length can intuitively reflect lower limb motor function and the improvement effect after mechanical assistance.	Yue et al. (2021) Mu (2022)
Step size	Test the average step length of athletes while walking, measured in cm, test three times, and take the average value		
Physical endurance	A 6-min walk test is used to measure the maximum distance an athlete can walk in 6 min, in meters. The test is conducted once, and the actual distance is recorded.	A 6-min walking distance is a classic indicator for assessing exercise endurance, cardiopulmonary function, and sustained exercise capacity, and it has been widely used in rehabilitation effectiveness evaluation studies.	Hu et al. (2022)
Sport accuracy	Use lower limb target positioning tests, have athletes wear the relevant equipment, complete lower limb movements at specified angles (such as knee flexion and hip extension), record the accuracy of the movements, in %, test 5 times, and take the average.	Movement accuracy can reflect an athlete’s control of actions, body balance, and task completion ability under assisted conditions, compensating for the inadequacy of simple gait indicators in reflecting the level of sports skills, making the evaluation system more comprehensive.	Huo et al. (2023)

(2) Referring to existing studies (Wang et al., 2026; Liu, 2022; Lu et al., 2022; Ouyang et al., 2026), standardized scales shown in Table 2 were selected for psychological state assessment to evaluate athletes’ self-efficacy, sports motivation, anxiety and depression. All scales have been tested for reliability and validity and are suitable for disabled athletes.

**Table 2:** Psychological state assessment scales.

Scale	Number of entries	Scoring method	Evaluation method	Source
Exercise Self-Efficacy Scale (SESS)	15	1–5	The higher the total score, the stronger the athlete’s exercise self-efficacy.	Wei et al. (2008)
Exercise Motivation Scale (SMS)	28	1–7	The higher the score in each dimension, the stronger the athlete’s motivation for exercise.	Pelletier et al. (1995)
Self-Rating Anxiety Scale (SAS)	20	1–4	The higher the total score, the more severe the athlete’s anxiety.	Zung (1971)
Self-Rating Depression Scale (SDS)	20	1–4	The higher the total score, the more severe the athlete’s depressive symptoms.	Zung (1965)

(3) Semi-structured interview: After the intervention, 15 athletes in the experimental group were interviewed semi-structurally. The interview outline mainly included: subjective feelings of training wearing lower limb exoskeleton robots; changes in psychological state during training (such as confidence, motivation, emotions, etc.); influence and feelings of exoskeleton robot training on their own athletic performance; whether they think exoskeleton robots affect athletic performance through psychological changes, and what specific aspects are manifested. Each interview lasted 15–20 min, with full recording. After the interview, transcription was performed to form qualitative data.

#### Data collection and processing

**Figure 1:**
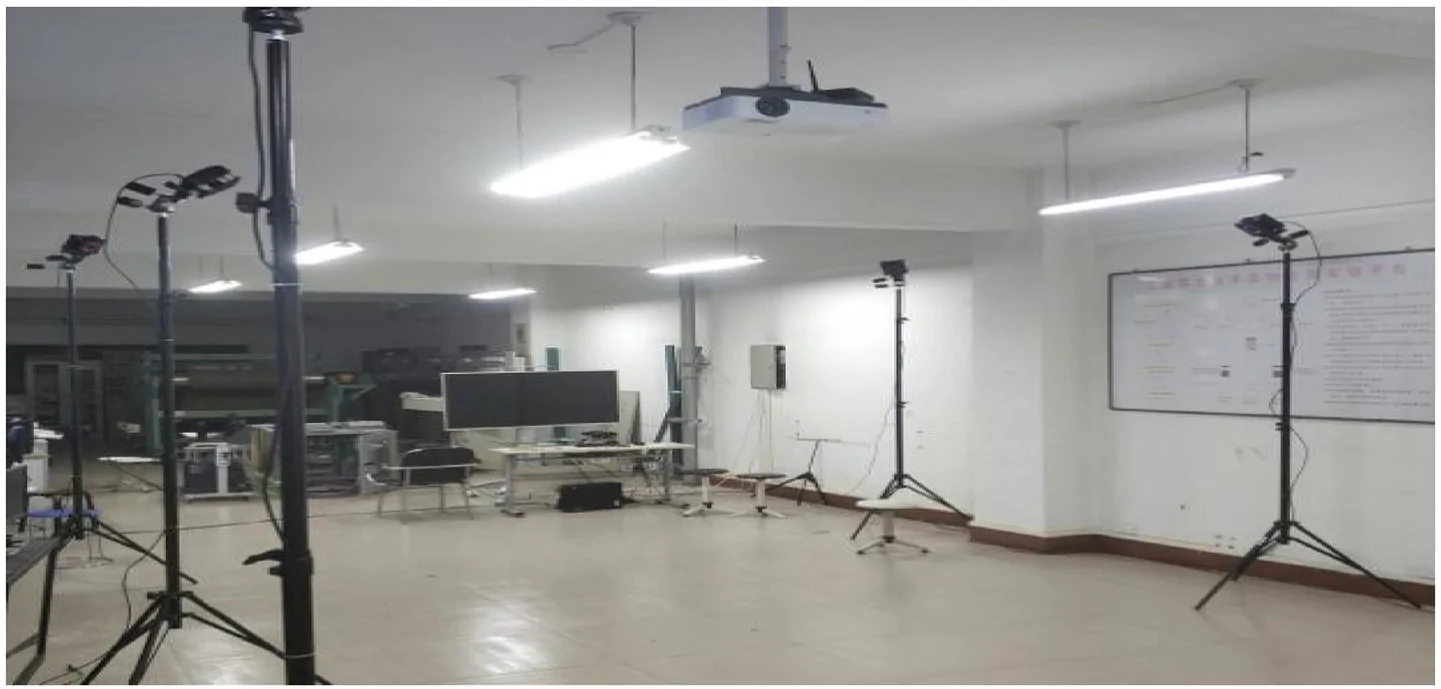
3D infrared passive optical motion capture system.

This article uses the CONTRX software and a three-dimensional infrared passive optical motion capture system, as shown in Figure 1, to collect gait data of the human body. First, two position sensors are placed on the hip, knee, and ankle joints of the lower limbs, respectively. Some of the collected information is sent out in real time in the form of analog signals, which are then converted from analog to digital. The motion information of the human body in the sensors is transmitted to the computer, and parameters during the motion process are calculated according to the received signals.

**Figure 2:**
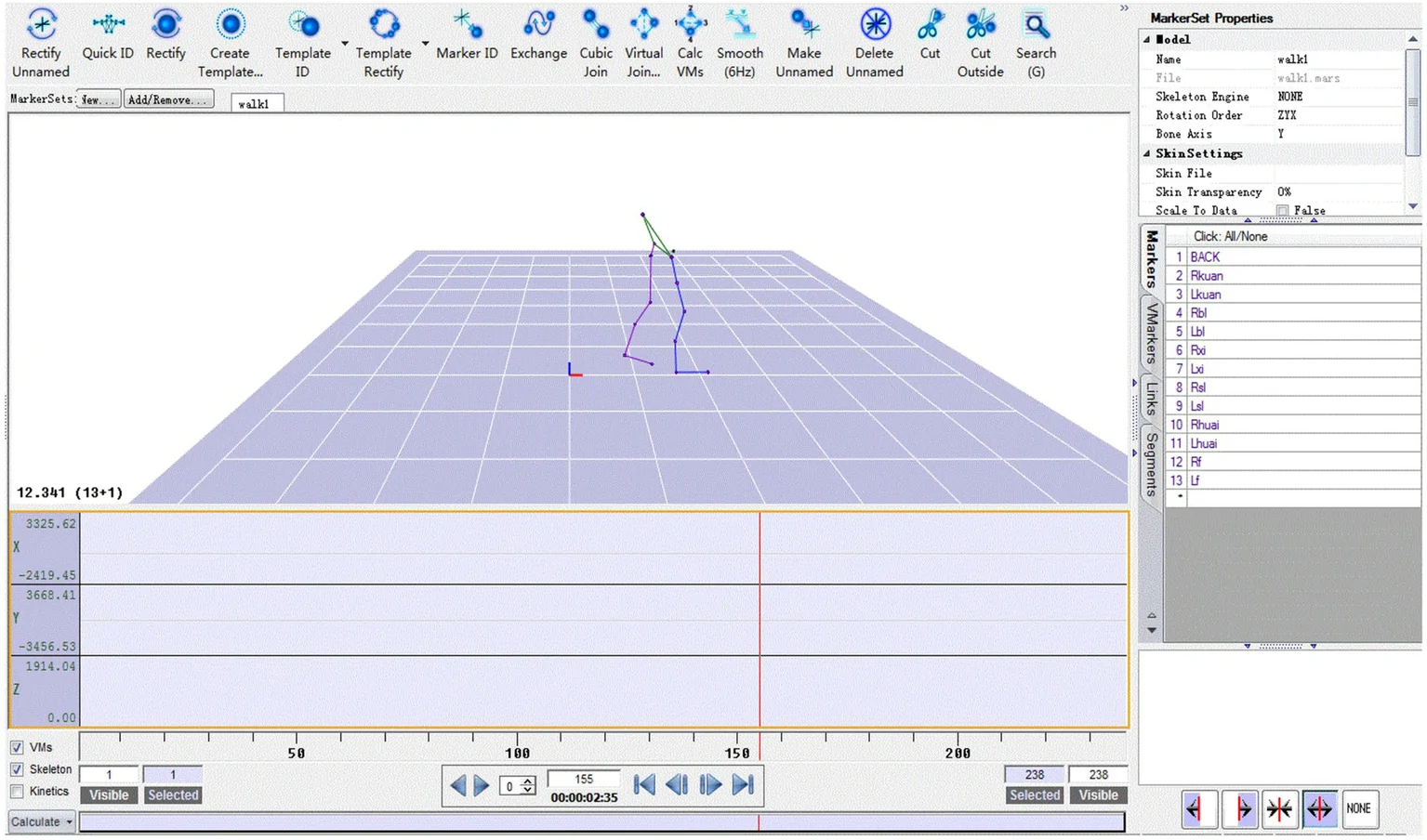
Screenshot of gait information during the collection process.

**Figure 3:**
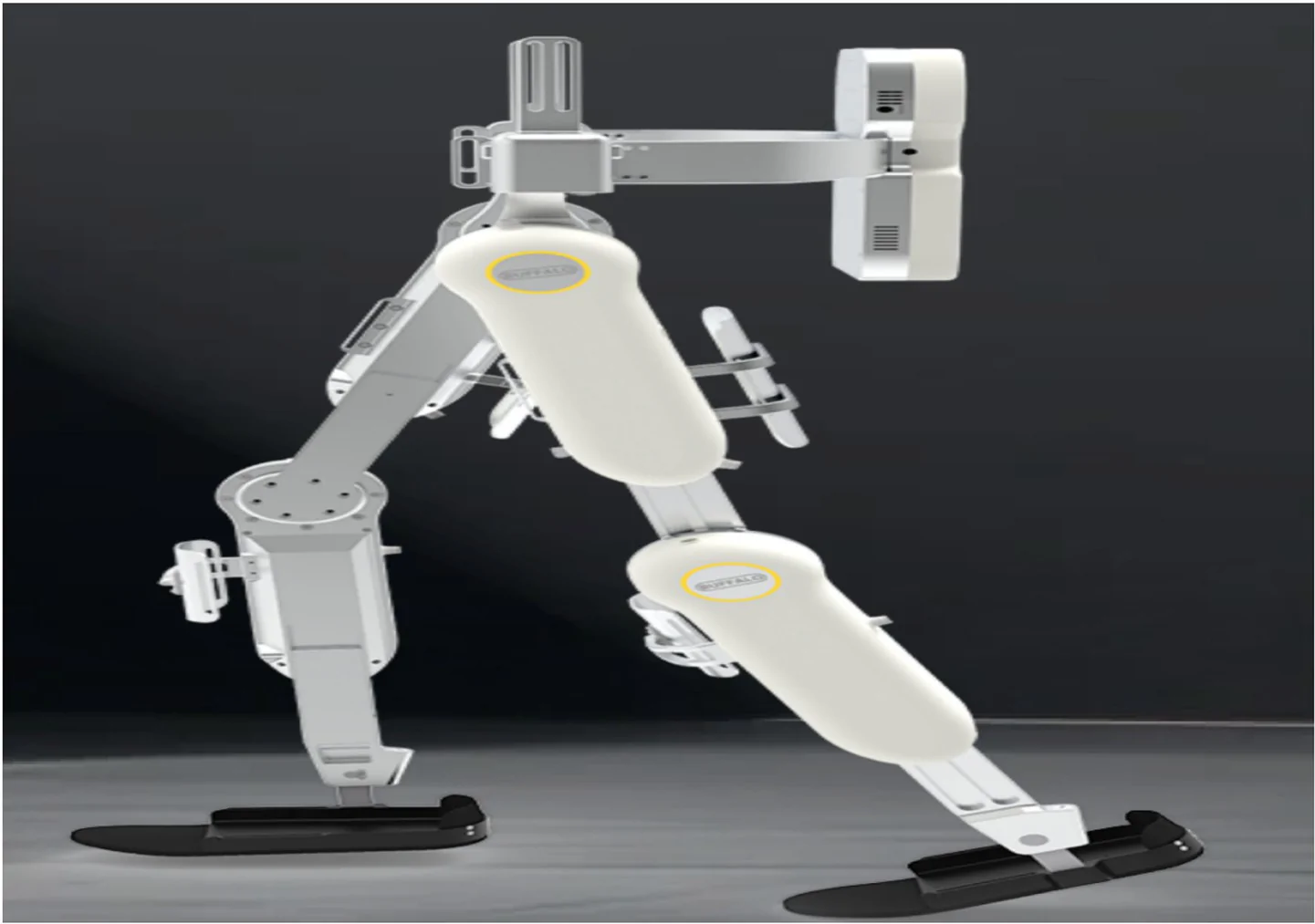
Aider3S robot.

SPSS 26.0 statistical software was used for data processing and analysis. Measurement data were expressed as (*x* ± s). Statistical analysis was done with Analysis of variance (ANOVA); Pearson correlation analysis was used to explore the correlation between psychological indicators and athletic performance indicators; mediating effect test (Bootstrap method) was used to verify the mediating role of psychological mechanisms, with a test level of *α* = 0.05. Thematic analysis was used for qualitative data to extract key themes, explore the action pathways of psychological mechanisms, and mutually verify with quantitative data to ensure the comprehensiveness and reliability of research conclusions (Figures 2, 3).

## Research results

### Comparison of athletic performance between the two groups before and after intervention

As shown in Table 3, before intervention, there were no significant differences in walking speed, step length, 6-min walking distance and sports accuracy between the two groups (*p* > 0.05), which were comparable; at the end of the 6th week of intervention, the walking speed, step length, 6-min walking distance and sports accuracy of the experimental group were higher than those of the control group, but the differences were only statistically significant in walking speed and step length (*p* < 0.05); at the end of the 12th week of intervention, all athletic performance indicators of the experimental group were significantly higher than those of the control group (*p* < 0.05), and all indicators of the experimental group after intervention were significantly higher than those before intervention (*p* < 0.05). Although the indicators of the control group after intervention were improved, the improvement range was significantly lower than that of the experimental group (*p* < 0.05).

**Table 3:** Comparison of athletic performance indicators of the two groups before and after the intervention.

Indicator	Effect type	*F*	df1, df2	*P*	Biased *η*^2^	Interaction effect 95% CI
Walking Speed (m/s)	Group main effect	18.925	1,28	∗∗ < 0.001	0.403	[0.167,0.582]
	Main effect of time	42.637	1.726,48.328	∗∗ < 0.001	0.603	[0.421,0.716]
	Group × Time Interaction	11.368	1.726,48.328	∗∗ < 0.001	0.289	[0.078,0.462]
Stride length (cm) (cm)	Group main effect	16.771	1,28	∗∗ < 0.001	0.373	[0.143,0.556]
	Main effect of time	38.152	1.681,47.068	∗∗ < 0.001	0.577	[0.390,0.698]
	Group × Time Interaction	9.842	1.681,47.068	∗∗ < 0.001	0.259	[0.062,0.432]
6-min walking distance (m)	Group main effect	14.306	1,28	∗∗ < 0.001	0.336	[0.112,0.524]
	Main effect of time	51.274	1.593,44.604	∗∗ < 0.001	0.647	[0.476,0.748]
	Group × Time Interaction	7.915	1.593,44.604	∗∗ < 0.01	0.220	[0.041,0.394]
Motor Accuracy (%)	Group main effect	17.453	1,28	∗∗ < 0.001	0.382	[0.151,0.564]
	Main effect of time	47.831	1.645,46.061	∗∗ < 0.001	0.630	[0.452,0.736]
	Group × Time Interaction	10.127	1.645,46.061	∗∗ < 0.001	0.265	[0.067,0.438]

** The difference is statistically very significant.

### Comparison of psychological state between the two groups before and after intervention

As shown in Table 4, before intervention, there were no significant differences in self-efficacy, intrinsic sports motivation, anxiety and depression scores between the two groups (*p* > 0.05), which were comparable; at the end of the 6th week of intervention, the self-efficacy and intrinsic sports motivation scores of the experimental group were significantly higher than those of the control group (*p* < 0.05), and the anxiety and depression scores were significantly lower than those of the control group (*p* < 0.05); at the end of the 12th week of intervention, the psychological state improvement effect of the experimental group was more significant. The self-efficacy and intrinsic sports motivation scores were significantly higher than those of the control group (*p* < 0.01), and the anxiety and depression scores were significantly lower than those of the control group (*p* < 0.01). All psychological indicators of the experimental group after intervention were significantly better than those before intervention (*p* < 0.01). Although the psychological indicators of the control group after intervention were improved, the improvement range was significantly lower than that of the experimental group (*p* < 0.05).

**Table 4:** Comparison of psychological state indicators of two groups of athletes before and after intervention.

Indicator	Effect type	*F*	df1, df2	*P*	Biased *η*^2^	Interaction effect 95% CI
Self-efficacy (score)	Group main effect	22.146	1,28	∗∗ < 0.001	0.440	[0.201,0.613]
	Main effect of time	56.319	1.612,45.136	∗∗ < 0.001	0.664	[0.501,0.760]
	Group × Time Interaction	13.524	1.612,45.136	∗∗ < 0.001	0.324	[0.103,0.494]
Intrinsic Motivation (points)	Group main effect	20.873	1,28	∗∗ < 0.001	0.423	[0.186,0.600]
	Main effect of time	53.682	1.605,44.940	∗∗ < 0.001	0.654	[0.487,0.753]
	Group × Time Interaction	12.467	1.605,44.940	∗∗ < 0.001	0.304	[0.089,0.474]
Anxiety (score)	Group main effect	19.551	1,28	∗∗ < 0.001	0.407	[0.171,0.586]
	Main effect of time	49.265	1.634,45.752	∗∗ < 0.001	0.638	[0.463,0.742]
	Group × Time Interaction	10.839	1.634,45.752	∗∗ < 0.001	0.277	[0.074,0.449]
Depression (score)	Group main effect	18.740	1,28	∗∗ < 0.001	0.396	[0.161,0.577]
	Main effect of time	46.137	1.658,46.424	∗∗ < 0.001	0.621	[0.440,0.730]
	Group × Time Interaction	9.653	1.658,46.424	∗∗ < 0.001	0.254	[0.058,0.427]

### Correlation analysis between psychological indicators and athletic performance indicators

Pearson correlation analysis results are shown in Table 5. At the end of the 12th week of intervention, athletes’ self-efficacy and intrinsic sports motivation were significantly positively correlated with walking speed, step length, 6-min walking distance and sports accuracy (*r* = 0.623–0.758, *p* < 0.01); anxiety and depression were significantly negatively correlated with all athletic performance indicators (*r* = −0.587—-0.712, *p* < 0.01). It indicates that the psychological state of disabled athletes is closely related to athletic performance: the better the psychological state, the better the athletic performance; the more serious the negative emotions, the worse the athletic performance.

**Table 5:** Correlation analysis between psychological indicators and athletic performance indicators (*r* value).

Psychological indicators	Statistic	Walking speed	Step size	6-min walking distance	Sport accuracy
Self-efficacy	*r*	0.725**	0.758**	0.689**	0.657**
	*p*	*p* < 0.001	*P* < 0.001	*P* < 0.001	*P* < 0.001
	95%CI	[0.494,0.861]	[0.547,0.878]	[0.437,0.841]	[0.389,0.823]
Intrinsic motivation	*r*	0.698**	0.713**	0.675**	0.623**
	*p*	*P* < 0.001	*P* < 0.001	*P* < 0.001	*p* < 0.001
	95%CI	[0.451,0.846]	[0.475,0.854]	[0.416,0.833]	[0.339,0.803]
Anxiety	*r*	−0.692**	−0.712**	−0.658**	−0.587**
	*p*	*P* < 0.001	*P* < 0.001	*P* < 0.001	*P* < 0.001
	95%CI	[−0.842,−0.442]	[−0.853,−0.473]	[−0.823,−0.390]	[−0.782,−0.288]
Depression	*r*	−0.679**	−0.695**	−0.634**	−0.602**
	*p*	*P* < 0.001	*p* < 0.001	*p* < 0.001	*p* < 0.001
	95%CI	[−0.835,−0.422]	[−0.844,−0.447]	[−0.809,−0.355]	[−0.791,−0.309]

**P < 0.01, the correlation is extremely statistically significant.

### Mediating effect test of psychological mechanisms

The Bootstrap method (5,000 repeated samplings) was used to test the mediating role of psychological mechanisms between lower limb exoskeleton robots and athletic performance of disabled athletes. The results are shown in Table 6:

**Table 6:** Mediating effect test results of psychological mechanisms.

Mediator variable	Direct effect value	Direct effect 95% CI	Mediator effect value	Mediation effect 95% CI	Proportion of the total effect accounted for by the mediating effect (%)	*P*
Self-efficacy	0.326	[0.213,0.440]	0.218	[0.136,0.305]	66.87	<0.01
Intrinsic motivation	0.312	[0.201,0.425]	0.195	[0.112,0.281]	62.50	<0.01
Anxiety	0.335	[0.224,0.448]	0.187	[0.104,0.273]	55.82	<0.01
Depression	0.329	[0.218,0.442]	0.179	[0.097,0.266]	54.41	<0.01

The mediating effect test adopts the Bootstrap method with 5,000 repeated samplings. *P* < 0.01 indicates that the mediating effect is extremely statistically significant.

Mediating effect of self-efficacy: The direct effect value of lower limb exoskeleton robot intervention on athletic performance was 0.326 (*p* < 0.01), and the mediating effect value through self-efficacy was 0.218 (*p* < 0.01), accounting for 66.87% of the total effect, indicating that self-efficacy plays a partial mediating role between lower limb exoskeleton robots and athletic performance.

Mediating effect of intrinsic sports motivation: The direct effect value of lower limb exoskeleton robot intervention on athletic performance was 0.312 (*p* < 0.01), and the mediating effect value through intrinsic sports motivation was 0.195 (*p* < 0.01), accounting for 62.50% of the total effect, indicating that intrinsic sports motivation plays a partial mediating role between lower limb exoskeleton robots and athletic performance.

Mediating effect of anxiety: The direct effect value of lower limb exoskeleton robot intervention on athletic performance was 0.335 (*p* < 0.01), and the mediating effect value through anxiety was 0.187 (*p* < 0.01), accounting for 55.82% of the total effect, indicating that anxiety plays a partial mediating role between lower limb exoskeleton robots and athletic performance.

Mediating effect of depression: The direct effect value of lower limb exoskeleton robot intervention on athletic performance was 0.329 (*p* < 0.01), and the mediating effect value through depression was 0.179 (*p* < 0.01), accounting for 54.41% of the total effect, indicating that depression plays a partial mediating role between lower limb exoskeleton robots and athletic performance.

In summary, self-efficacy, intrinsic sports motivation, anxiety and depression together constitute the psychological mechanism for lower limb exoskeleton robots to improve the athletic performance of disabled athletes, all playing a partial mediating role, verifying the research hypothesis H3.

### Qualitative interview result analysis

The themes of semi-structured interviews with 15 athletes in the experimental group are shown in Table 7. The interview results further confirm the action pathways of psychological mechanisms.

**Table 7:** Core themes and frequencies of qualitative interviews.

Core theme	Number of people mentioned (*n* = 15)	Core statement
Reduce exercise frustration	13	After wearing the exoskeleton, the fluidity of movements improves, the experience of training failures is reduced, and the courage to persist in training is enhanced.
Enhance body control	14	The exoskeleton provides stable support, helps autonomously control lower limb movements, reduces the feeling of loss of control, and improves confidence in movement.
Enhance self-identity	12	Break through physiological limitations, approach the athletic level of able-bodied athletes, and get rid of the negative perception that ‘disability = low ability’.
Relieve mental stress	10	Improved athletic performance, relief from training stress and competition anxiety, reduced feelings of inferiority, and a calmer mindset

#### Interview procedure

Interview Timing: Conducted within 3 days after the 12-week intervention is fully completed and the final quantitative test is done; held in an independent, quiet rehabilitation interview room with no third parties present; participants are informed in advance about the use of recordings, anonymous transcription, and their right to stop speaking at any time; each interview lasts 15–20 min, recorded in stereo; a paper outline is provided before the interview, allowing athletes to freely add extra psychological experiences.

Unified interview outline with four core modules: ① Adaptation to wearing the exoskeleton and movement experience; ② Confidence, motivation, and emotional dynamics throughout the training; ③ How mechanical assistance changes training behaviors and competition expectations; ④ How perceived psychological changes directly affect the quality of sports movements.

#### Transcription and coding process

Step 1: The research assistant transcribed all 15 recordings word for word, removing names, locations, and other identifying information, and replaced respondents with labels T1–T15.

Step 2: Two people independently did open coding (two coders with qualifications in sports psychology, not sharing the original transcripts), extracting initial concepts and short units of text.

Step 3: Checked coding consistency, Kappa coefficient = 0.81, showing good agreement; any disagreements were discussed in a team meeting to reach a consensus.

Step 4: Axial coding aggregated primary concepts into secondary categories; selective coding distilled four main themes.

Step 5: Triangulation: cross-checked quantitative results, interview themes, and athletes’ original quotes to ensure the reliability of the qualitative conclusions.

### Lower limb exoskeleton robot-assisted training helps reduce sports frustration

Thirteen athletes stated that after wearing exoskeleton robots, they could easily complete movements such as walking and limb stretching, reducing failure experiences in training and significantly lowering frustration. In previous conventional training, due to lower limb function limitations, it was difficult to achieve expected sports goals, often leading to frustration and even wanting to give up training. For example: “It was difficult to complete a simple stepping movement before, often making mistakes, and the more I practiced, the less confident I became. After wearing the exoskeleton, movements become smooth with few mistakes, and I gradually have the courage to persist.”

Representative quotes: Representative Quote 1 (T3, Spinal Cord Injury Track and Field Athlete): “Previously, every time I practiced straight-line walking, I would lose balance repeatedly and fall. After 10 min, I would collapse, feeling I would never meet the training standards; After wearing the exoskeleton, your gait stabilizes, you will not lose control even after several 100 m in a row, and your chances of failure are greatly reduced, so you do not have to give up right after training.”

Representative Citation 2 (T9, table tennis player with lower limb amputation): In conventional rehabilitation, it is difficult to complete directional movement using only one’s own strength, leading to long-term accumulation of weakness; With the help of an exoskeleton, you can complete the entire set of movement training, and your sense of frustration is significantly reduced.

### Lower limb exoskeleton robot-assisted training helps enhance body control

Fourteen athletes stated that exoskeleton robots can provide stable assistance, help them better control lower limb movements, reduce a sense of loss of control in sports, and thus enhance confidence in their sports ability. In previous conventional training, balance was reduced due to lower limb injury, making it impossible to accurately control the body. For example: “I used to wobble when walking, worried about falling, and could not control my body well. After wearing the exoskeleton, I feel support for my lower limbs and can control my steps independently. This sense of control makes me dare to try more complex sports movements,” which is closely related to the precise assistance function of exoskeleton robots (Wang et al., 2025).

Representative quotes: Quote (T12): “Without exoskeleton support, my lower limb strength was completely uncontrollable, constantly worried about falling; The device provides balanced power support, with fully controllable rhythm of hip and knee movement. This sense of body control is something regular training cannot provide.”

### Lower limb exoskeleton robot-assisted training helps improve self-identity

Twelve athletes stated that exoskeleton robots helped them break through physical limitations, complete previously unattainable sports movements, even approaching the sports level of able-bodied athletes, thereby improving self-identity and getting rid of the negative cognition that “disability equals low ability.” In previous conventional training, they often dared not try some difficult movements due to physical disability. For example: “I used to think that because of my disability, I could never train like able-bodied athletes. After wearing the exoskeleton, I can complete similar movements to them, feel like a qualified athlete, and my self-confidence has been greatly improved,” which is consistent with the conclusion of existing studies on exoskeleton robots improving self-identity of disabled people (Meng, 2025).

Representative quotes: Quote (T5): “Previously, accepting disability meant it could not keep up with the training intensity of able-bodied athletes, leading to long-term self-denial; The exoskeleton allows me to perform equally difficult movements, no longer defining myself as a ‘disabled person with limited ability,’ and truly accepting my identity as an athlete.”

### Lower limb exoskeleton robot-assisted training helps relieve psychological pressure

Ten athletes stated that long-term training pressure, worry about competition results and psychological burden caused by physical disability often put them in a state of anxiety and depression; after wearing exoskeleton robots, the improvement of athletic performance gave them hope for progress, and psychological pressure was effectively relieved. “The better training effect makes me more confident about the competition, no longer as anxious as before, no longer feel inferior because of my disability, and my mindset has become more peaceful.”

Representative quotes: Quote (T7): “Before the race, I was always worried that my mobility shortcomings would drag me down my results, leading to long-term insomnia and anxiety; After 12 weeks of exoskeleton training, walking and endurance improved significantly, fear of competition greatly decreased, and feelings of inferiority and suppression greatly lessened.”

Supplementary coding explanation: The four major themes are generated by two independent coding units, with a total of 217 original valid coding units, including 62 reductions in frustration, 78 on bodily control, 45 on self-identification, and 32 on stress relief, with quantitative frequency supporting the reliability of the themes.

## Discussion

### Improvement effect of lower limb exoskeleton robots on athletic performance of disabled athletes

After 12 weeks of intervention, the values and improvement ranges of athletic performance indicators of athletes in the experimental group were significantly higher than those in the control group, indicating that lower limb exoskeleton robots can effectively improve the athletic performance of disabled athletes, verifying hypothesis H1.

Lower limb exoskeleton robots can compensate for physical defects such as insufficient lower limb muscle strength and limited joint mobility of disabled athletes through mechanical structures, provide stable support and power, and help athletes complete movements more standardized and efficiently. Meanwhile, the anthropomorphic gait design of exoskeleton robots can help athletes correct stepping posture, rebuild walking memory of nerves and muscles, and improve sports coordination and accuracy (Lorenzutti et al., 2025). In addition, exoskeleton robots can reduce energy consumption during exercise and muscle fatigue, thereby improving sports endurance and providing a physiological foundation for the improvement of athletic performance (Pinto-Fernandez et al., 2020). This is consistent with the findings of Qiu et al. (2024).

However, at the end of the 6th week of intervention, only two indicators (walking speed and step length) in the experimental group were significantly higher than those in the control group, while the differences in sports endurance and sports accuracy were not significant, indicating that the improvement of athletic performance of disabled athletes by lower limb exoskeleton robots requires a certain intervention cycle to achieve comprehensive effects. This is because athletes need time to adapt to the wearing and assistance mode of exoskeleton robots, gradually establish human-machine collaborative sports habits, and the improvement of physiological functions and sports skills also requires long-term training accumulation (Babič et al., 2021).

This study only included 30 city-level disabled athletes, so the sample size is small. The conclusions can only represent people with moderate lower limb dysfunction who have over a year of training experience, and cannot be directly applied to those with severe disabilities, beginners in rehabilitation, or middle-aged and elderly disabled groups. The results from the 12-week short-term intervention only show short-term training effects, and the long-term outcomes still need to be verified. The study’s conclusions have some preliminary exploratory value and should not be overgeneralized.

### Improvement effect of lower limb exoskeleton robots on psychological state of disabled athletes

After intervention, the self-efficacy and intrinsic sports motivation of athletes in the experimental group were significantly improved, anxiety and depression were significantly relieved, and the improvement effect was significantly better than that in the control group, verifying research hypothesis H2.

This is because lower limb exoskeleton robots help athletes break through physical limitations and complete previously unattainable sports movements, enhance confidence in sports by feeling the improvement of their sports ability, and improve self-efficacy. As stated by athletes in the interview, the sense of body control brought by exoskeleton robots enhances recognition of their sports ability; the gradual improvement of athletic performance allows athletes to see the effect of training, stimulates their intrinsic sports motivation, changes from passive training to active training. The improvement of intrinsic motivation prompts athletes to invest more energy in training, forming a virtuous circle of training effect improvement → sports motivation enhancement → athletic performance improvement; the assistance of exoskeleton robots reduces training difficulty and sports frustration, thereby relieving anxiety and depression caused by long-term physical disorders and establishing a positive psychological state. In addition, as an advanced rehabilitation device, exoskeleton robots themselves can make athletes feel social attention and support for disabled athletes, further relieving psychological pressure and improving psychological adaptability. This is consistent with the findings of Rodríguez-Fernández et al. (2021).

For the control group, although conventional rehabilitation training can improve athletes’ physiological functions to a certain extent, it is difficult to break through physical limitations, cannot effectively relieve sports frustration, and cannot quickly stimulate intrinsic sports motivation, so the improvement effect on psychological state is limited. This further indicates that lower limb exoskeleton robots are not only a physical rehabilitation tool but also an effective psychological intervention carrier, which can achieve synchronous improvement of psychological state through physical assistance.

This study only selected four core psychological indicators and did not include variables like mental resilience, bodily self-esteem, or fear of movement. It can only explain part of the psychological change paths and cannot fully cover all psychological aspects of athletes with disabilities, so the conclusions have limitations.

### Mediating role of psychological mechanisms between lower limb exoskeleton robots and athletic performance

Through correlation analysis and mediating effect test, it is confirmed that self-efficacy, intrinsic sports motivation, anxiety and depression all play a partial mediating role between lower limb exoskeleton robots and athletic performance of disabled athletes, verifying research hypothesis H3 and revealing the psychological path of exoskeleton robots improving athletic performance.

From the specific role of mediating effect, the mediating effect proportion of self-efficacy is the highest (66.87%), indicating that exoskeleton robots mainly indirectly promote athletic performance by improving athletes’ self-efficacy. As an individual’s cognition and confidence in his ability, self-efficacy directly affects sports decision-making and effort level. Disabled athletes with improved self-efficacy dare to challenge more difficult movements in training and are more persistent in the face of difficulties, thereby improving athletic performance. Followed by intrinsic sports motivation (62.50%), the stimulation of intrinsic motivation allows athletes to gain fun and a sense of achievement from training itself, take the initiative to invest more energy in improving sports skills, rather than simply completing training tasks. Intrinsic motivation is an important driving force for athletic performance improvement. The mediating effect proportions of anxiety and depression are 55.82 and 54.41% respectively, indicating that exoskeleton robots indirectly improve athletic performance by relieving negative emotions and reducing their inhibition on athletic performance. Existing studies have shown that negative emotions such as anxiety and depression affect athletes’ attention, reaction speed and sports coordination, leading to decreased athletic performance (Anderson et al., 2025). By improving athletes’ psychological state and relieving negative emotions, exoskeleton robots can help them maintain a good psychological state in training and competitions and give full play to their sports ability. This is consistent with the findings of Lu et al. (2024).

In addition, qualitative interview results further confirm the mediating action pathways. The four core themes of “reducing sports frustration, enhancing body control, improving self-identity, relieving psychological pressure” are all closely related to the improvement of self-efficacy, sports motivation and emotional state, indicating that exoskeleton robots gradually improve athletes’ psychological state through these four dimensions of psychological experience, thereby promoting athletic performance. This also shows that the mediating role of psychological mechanisms is not a single-dimensional role, but a result of the synergistic effect of multiple psychological factors, which together constitute the psychological path for exoskeleton robots to improve athletic performance.

The mediation effect was only found in this small sample with repeated measurements and has not been replicated in larger, multi-center cohorts. The strength of the mediation effect might vary across different disability types (spinal cord injury/amputation), but we did not do subgroup analyses, so the conclusions about the mechanism are just preliminary evidence.

## Conclusion and Prospect

### Conclusion

Through lower limb exoskeleton robot intervention and randomized controlled trial design, empirical research finds that: (1) Lower limb exoskeleton robots can significantly improve the athletic performance of disabled athletes in terms of walking speed, step length, sports endurance and sports accuracy, and this improvement effect has phased characteristics, requiring long-term intervention to achieve comprehensive results; (2) Lower limb exoskeleton robot intervention training can significantly improve the psychological state of disabled athletes, improve self-efficacy and intrinsic sports motivation, and relieve negative emotions such as anxiety and depression; (3) The improvement of psychological state can indirectly promote athletic performance, and psychological mechanisms play a partial mediating role in the process of lower limb exoskeleton robots promoting athletic performance improvement of disabled athletes.

### Research limitations and future research directions

There are still some limitations in the study: (1) the sample size of research subjects is small, and only municipal and above disabled athletes are selected, so the universality of the results is limited. The sample size can be expanded in the future; (2) The intervention period is 12 weeks, and no long-term follow-up is conducted on athletes, so it is impossible to clarify the long-term impact of exoskeleton robots on athletic performance and psychological state. The intervention period can be extended to explore its long-term effect in the future; (3) The study explores the mediating role of four psychological factors: self-efficacy, sports motivation, anxiety and depression, without considering the impact of other psychological factors. More psychological variables can be included in the future; (4) The exoskeleton robot used in this study is a single model. There are differences in technical parameters and assistance modes of different models of exoskeleton robots, which may affect the research results. The intervention effects of different models of exoskeleton robots can be compared in the future.

Future research can be carried out from the following directions: (1) Combine artificial intelligence technology to optimize the personalized adaptation mode of exoskeleton robots, and formulate personalized training programs according to athletes’ disability types, sports needs and psychological characteristics; (2) In-depth explore the integration path of psychological intervention and exoskeleton robot technical intervention, and build an integrated training system of “technical assistance + psychological guidance” to further improve the intervention effect; (3) Expand the research scope, extend the application of exoskeleton robots to disabled athletes of different ages and sports, and explore the application value in the rehabilitation of ordinary disabled people to promote the widespread popularization of technology.

## Data Availability

The original contributions presented in the study are included in the article/supplementary material, further inquiries can be directed to the corresponding author.
